# Microcrystalline Tyrosine and Aluminum as Adjuvants in Allergen-Specific Immunotherapy Protect from IgE-Mediated Reactivity in Mouse Models and Act Independently of Inflammasome and TLR Signaling

**DOI:** 10.4049/jimmunol.1800035

**Published:** 2018-03-28

**Authors:** Deborah S. Leuthard, Agathe Duda, Sandra N. Freiberger, Sina Weiss, Isabella Dommann, Gabriele Fenini, Emmanuel Contassot, Matthias F. Kramer, Murray A. Skinner, Thomas M. Kündig, Matthew D. Heath, Pål Johansen

**Affiliations:** * Department of Dermatology, University of Zurich, 8091 Zurich, Switzerland;; † Department of Dermatology, University Hospital Zurich, 8091 Zurich, Switzerland;; ‡ Bencard Allergie GmbH, 80992 Munich, Germany; and; § Allergy Therapeutics Ltd., Worthing BN14 8SA, United Kingdom

## Abstract

Allergen immunotherapy (AIT) is the only modality that can modify immune responses to allergen exposure, but therapeutic coverage is low. One strategy to improve AIT safety and efficacy is the use of new or improved adjuvants. This study investigates immune responses produced by microcrystalline tyrosine (MCT)–based vaccines as compared with conventional aluminum hydroxide (alum). Wild-type, immune-signaling–deficient, and TCR-transgenic mice were treated with different Ags (e.g., OVA and cat dander Fel d 1), plus MCT or alum as depot adjuvants. Specific Ab responses in serum were measured by ELISA, whereas cytokine secretion was measured both in culture supernatants by ELISA or by flow cytometry of spleen cells. Upon initiation of AIT in allergic mice, body temperature and further clinical signs were used as indicators for anaphylaxis. Overall, MCT and alum induced comparable B and T cell responses, which were independent of TLR signaling. Alum induced stronger IgE and IL-4 secretion than MCT. MCT and alum induced caspase-dependent IL-1β secretion in human monocytes in vitro, but inflammasome activation had no functional effect on inflammatory and Ab responses measured in vivo. In sensitized mice, AIT with MCT-adjuvanted allergens caused fewer anaphylactic reactions compared with alum-adjuvanted allergens. As depot adjuvants, MCT and alum are comparably effective in strength and mechanism of Ag-specific IgG induction and induction of T cell responses. The biocompatible and biodegradable MCT seems therefore a suitable alternative adjuvant to alum-based vaccines and AIT.

## Introduction

Allergy is a leading cause of chronic illness (1), with social and economic impacts on life quality and health care costs (2). Allergen immunotherapy (AIT) is disease-modifying and reduces symptoms and medication use in allergic rhinoconjunctivitis and asthma (3), but because of ignorance of efficacy, potential side effects, and a long treatment duration, <10% of allergy patients chose to receive AIT (4). Hence, safer or more effective AIT using alternate administration routes, better allergens, and better adjuvants (5) is needed.

Adjuvants enhance Ag-specific immune responses in vaccines and AIT. The most used adjuvant is aluminum hydroxide (alum), which was introduced empirically in vaccines a century ago (6). Suggested mechanisms of action are depot formation (7), enhancement of Ag uptake by APCs (8), and NLRP3 inflammasome activation (9–13). In animals and humans, alum mediates a Th2 immune response (7), which counteracts recognized therapeutic mechanisms of AIT. Furthermore, there are concerns with respect to alum accumulation in tissues in AIT (14). Although alum has remained the adjuvant of choice across the broader vaccine scope, the nonessential amino acid l-tyrosine was developed as an alternative depot platform for delivering AIT as well as other vaccine Ags. This proprietary depot adjuvant is now referred to as microcrystalline tyrosine (MCT), which reflects its physicochemical properties, such as a distinct crystalline particle size and needle-like morphology. During processing steps, MCT is either coprecipitated with the candidate Ag or the Ag is adsorbed to the preformed MCT. MCT products are marketed for use in AIT, some of which are licensed (15), and MCT has been shown to facilitate allergen-specific IgG4 Ab production as well as IL-10 secretion from T cells (16–19). This study compares MCT and alum in mouse models to better understand their mode of action as adjuvants in AIT and other vaccines.

## Materials and Methods

### Materials

Grade V OVA and low-endotoxin OVA were from Sigma-Aldrich (Buchs, Switzerland). Alhydrogel 2% gel (alum) was from Brenntag (Fredrikssund, Denmark). Cat fur allergen extract was purchased from Stallergenes AG (Dietlikon, Switzerland). Birch pollen allergen extract (440 μg/ml protein, from 24.8% Bet v 1) and MCT (40 mg/ml) was provided by Allergy Therapeutics (Worthing, U.K.). Recombinant Fel d 1, the major cat fur allergen, was kindly provided by F. Thoms from the University of Zurich. Because allergen extracts are not rigorously standardized, we cannot compare how amounts of extracts in different units (IU, IR) compare with allergen content when comparing extracts from different sources.

### Animals

BALB/c (H2d) and C57BL/6 (H2b) mice were purchased from Envigo (Horst, the Netherlands). All gene-modified mice were bred at the Biologisches Zentrallabor of the University Hospital Zurich (Zurich, Switzerland) or at the Laboratory for Animal Science at the University of Zurich. Mice deficient in either MyD88, TIR (Toll/IL-1R) domain-containing adapter inducing IFN-β (TRIF), TLR4, or NALP3 were obtained from the Swiss Immunological Mutant Mouse Repository. Mice deficient for NALP3 and ASC (20) were originally from J. Tschopp (Biochemistry Institute, University of Lausanne, Switzerland) (21) and Genentech (San Francisco, CA), respectively (21). RAG1-deficient CD8 TCR transgenic OT-I mice were originally from Taconic Biosciences (Ry, Denmark). CD4 TCR transgenic OT-II mice were obtained from the Swiss Immunological Mutant Mouse Repository. All animals were kept and the experiments performed under specific pathogen-free conditions. The experiments were authorized by the cantonal veterinary office of Zurich and performed in accordance with Swiss animal law and regulations (animal experiment authorization numbers: ZH200/2014 and ZH 52/2016).

### Vaccine preparations

MCT vaccines were prepared according to an experimental protocol from Allergy Therapeutics. MCT or alum was rigorously vortexed (1 min) at room temperature to resuspend the particles. The required volumes of adjuvant and PBS were then transferred into sterile Ag-containing Eppendorf vials. The mixtures were again vortexed and left for 1 h at room temperature for complete protein adsorption.

### Immunization

Mice were immunized with vaccine preparations by injection of 100 μl s.c. (scruff of the neck) or i.p. Prior to each injection, vaccine preparations were thoroughly mixed. The vaccines were composed of OVA adsorbed to MCT or alum. Control mice received soluble OVA or PBS or were left untreated. Immunization doses were 0.01–100 μg of OVA, 0.27–2.7 mg of MCT, and 0.1–1 mg of alum. The number of immunization sessions was one to three and the interval between two sessions was 14 d if not otherwise specified.

Mice were tail bled, and the collected sera were analyzed by ELISA for OVA-specific Abs. Spleens were harvested at the end of the experiments and analyzed for OVA-specific CD8 T cell proliferation or cytokine production by flow cytometry or ELISA after in vitro restimulation with OVA.

### Adoptive T cell transfer and testing of CD4 and CD8 T cell responses

C57BL/6 mice received an i.v. transfer of 5 × 10^5^ OT-I and 10^6^ OT-II cells through the tail vein. One and eight days later, the mice were immunized s.c. with 100 μg OVA with MCT or alum. On day 15, spleens were isolated for further analysis of OVA-specific T cell responses by flow cytometry and ELISA.

### Analysis of serum Abs by ELISA

Sera were analyzed for Ag-specific Abs by ELISA (22–24). Briefly, 96-well plates were coated with OVA (4 μg/ml), cat fur allergen extract (1 IR/ml), recombinant Fel d 1 (1 μg/ml), or birch pollen allergen extract (30 μg/ml) in a NaHCO_3_/Na_2_CO_3_ buffer at pH 9.4 for IgG analysis. For IgE, the plates were coated with anti-IgE mAb (Bio-Rad Laboratories, Cressier, Switzerland). After overnight incubation at 4°C and blocking with 2.5% nonfat dried skimmed cow milk in PBS–Tween 20, sera diluted in PBS–Tween 20 were added and incubated for 2 h. After washing the plate, biotinylated anti-murine Abs (BD Pharmingen, Basel, Switzerland) were added for IgG analysis, and biotinylated Ag was added for IgE analysis. The plates were then incubated with streptavidin-conjugated HRP (BioLegend, Koblenz, Germany) and developed with tetramethylbenzidine substrate (BioLegend). Absorbance was read at 450 nm using a BioTek plate reader. The Ab titers were defined as the last serum dilution giving an ELISA OD higher than the mean of naive sera plus thrice the SD of the mean.

### Restimulation of splenocytes in vitro for analysis of cytokine secretion

Spleens were harvested and crushed through a cell strainer. After lysis of erythrocytes in RBC lysis buffer (Sigma), splenocytes were prepared at 5 × 10^5^ cells/well in RPMI-1640 complemented with FBS, glutamine, and antibiotics. The cultures were incubated with Ag at 37°C for 20 (IL-2, TNF-α) or 96 (IL-4, IL-10, IFN-γ) h. Cytokine secretion into the supernatant was measured by Ready-SET-Go! ELISA according to the manufacturer (Invitrogen, distributed by Thermo Fisher Scientific, Zug, Switzerland).

### Flow cytometry for analysis of T cell responses

Splenocytes were prepared as described above. For analysis of SIINFEKL-specific CD8 T cell proliferation, the cells were Fc receptor blocked with anti-CD16/32 and stained with fluorescent anti-CD8 and anti-CD44 Abs and PE-labeled H2K^b^-SIINFEKL (ProImmune, Oxford, U.K.). For analysis of intracellular cytokine production, the cells were incubated overnight with 10 μg/ml OVA and 2.5 μg/ml brefeldin A (Sigma) for the last 4 h. After washing and Fc receptor blocking, the cells were stained with anti-CD4, anti-CD8, and anti-CD44; fixed in 4% paraformaldehyde; permeabilized in Perm/Wash (BD Biosciences); and stained with anti–IFN-γ and anti–TNF-α Abs. All stainings were done in cold PBS supplemented with 2% FBS and protected from light. All staining Abs were from eBioscience or BD Pharmingen. The stained cells were acquired on BD FACSCanto II (BD Biosciences) and analyzed with FlowJo (FlowJo, Ashland, OR).

### Allergen immunotherapy

In a model of allergic anaphylaxis, mice were sensitized by four weekly i.p. injections of 1.0 IR units of cat dander allergen adsorbed to 0.5 mg alum (25, 26). Four weeks after the last injection, mice received AIT by three fortnightly s.c. injections of 25 μg recombinant cat (*Felis domesticus*) major allergen Fel d 1 adsorbed to either 2 mg MCT or 1 mg of alum. The range of adjuvant concentrations in all experiments was based on concentrations in clinical use. A control group was sensitized only. Mice were tail bled at different time points, and sera were analyzed for allergen-specific Abs.

Alternatively, mice were sensitized by i.p. injections of 5.8 μg birch pollen allergen extract on 0.5 mg alum. Starting 2 wk after the last sensitization, mice received AIT consisting of three fortnightly or five weekly s.c. injections of birch pollen allergen extract adsorbed to 2.0 mg MCT. The cumulative dose of birch pollen allergen extract protein was 264 μg (from 65.47 μg major allergen Bet v 1)—i.e., 3 × 88 μg (fortnightly) or 5 × 52.8 μg (weekly). One group of mice received weekly dose-incrementing AIT (10, 20, 40, 84, and 110 μg). Control mice were sensitized only.

### Allergen provocation test for analysis of anaphylaxis

Three weeks after the last AIT session, mice were challenged by an i.p. injection of 10 IR cat dander allergen extract (or 52.4 μg birch pollen allergen extract) to measure AIT-mediated protection against anaphylaxis. Before the challenge, and at 20–30 min intervals thereafter, body temperature was measured rectally with a digital Thermalert TH-5 thermometer with a RET-3 probe (Physitemp, Huron, NJ). A time–temperature curve was plotted and the area under curve (AUC) was calculated in GraphPad Prism v7.02 from GraphPad Software (La Jolla, CA). One day after the challenge, mice were euthanized and spleens were harvested for restimulation of splenocytes and analysis of cytokine secretion.

### In vitro assessment of inflammasome activation by MCT in human monocytes (THP-1)

THP-1 cells were pretreated with 3 μM PMA for 3 d followed by 24 h of incubation with 50 ng/ml ultra-pure LPS. The cells were then treated for 6 h with different doses of MCT or MCT plus zVAD. The concentration of IL-1β was measured in the culture supernatants by ELISA (Invitrogen). To determine the percentage of cell death, a lactate dehydrogenase cytotoxicity assay was used as described by the manufacturer (Pierce; Thermo Fisher Scientific). The proform and the mature form of IL-1β were separated by Western blot, and nigericin was used as positive control.

### Analysis of local and systemic inflammatory responses upon injection of adjuvants

Mice were injected i.p. with PBS, alum (1 mg), or MCT (2 mg) in 100 μl PBS. After the injection, a peritoneal lavage was done. Peritoneal cells were analyzed by flow cytometry using fluorescent Abs against CD11b, CD4, CD8, and B220.

### Statistics

Statistical analysis was done with GraphPad Prism v7.02. Two treatment groups were compared using nonparametric, two-tailed Mann–Whitney *U* tests. More groups were compared using Kruskal–Wallis tests with Dunn’s post hoc test for multiple comparisons. Time–temperature curves were integrated using the baseline temperature for the calculation of the AUC. Two-way parametric ANOVA was applied to test the statistical differences between the different treatment groups. Prior to this, normally distributed data were confirmed using the D’Agostino and Pearson normality test. Significant differences were annotated with asterisks: *p < 0.05, **p < 0.01.

## Results

### MCT enhances B and T cell responses in mice

**FIGURE 1.**
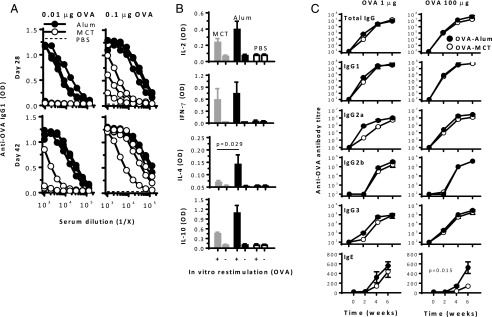
Immunogenicity testing of tyrosine- and alum-based vaccines in BALB/c mice. (**A**) Mice (*n* = 3–4) were injected with 0.01 or 0.1 μg OVA on alum (black symbols), MCT (open symbols), or in PBS (dashed lines) on days 0 and 28, and Ab titers were measured on days 28 and 42. Titration curves for individual mice are illustrated. (**B**) After a third injection with OVA 0.1 μg on day 56, splenocytes were harvested on day 62 and restimulated with OVA for analysis of cytokine secretion (mean + SD). (**C**) Mice (*n* = 5) were immunized with 1 (left) or 100 (right) μg OVA on days, 0, 14, and 28; total IgG, IgG1, IgG2a, IgG2b, IgG3, and IgE Abs were determined (mean ± SEM). The latter experiment is representative of three independent experiments with comparable results.

To measure the threshold for stimulation of Ag-specific Ab responses, mice were first immunized with low doses of OVA. After a single injection of 0.01 μg OVA, Ag-specific IgG1 Ab responses were observed in all mice that received OVA-alum (Fig. 1A), whereas no response was measured after 0.01 μg OVA-MCT or OVA-PBS. Upon a booster immunization on day 28, OVA-specific IgG1 was observed within 2 wk in one out of three mice receiving OVA-MCT. At 0.1 μg OVA, Ag-specific IgG1 was observed in all mice that received a single injection of either OVA-alum or OVA-MCT but not in the adjuvant-free OVA-PBS control. Similar results were observed for OVA-specific IgG2a (data not shown).

Mice that received 0.1 μg OVA were given a second booster immunization and were euthanized 6 d later for Ag restimulation of splenocytes in culture. Adjuvanted vaccines enhanced IL-2, IFN-γ, IL-4, and IL-10 cytokine secretion, with significantly higher IL-4 secretion in cells from alum-treated mice (Fig. 1B). Cells from mice immunized with OVA-PBS did not produce cytokines. The reduction of the MCT adjuvant dose from 2 to 0.4% did not affect the IgG1 responses notably (data not shown), but an earlier onset of IgG2a production was observed for the highest MCT dose; hence, 2% MCT was used in the subsequent studies.

Mice were then immunized thrice with 1 or 100 μg OVA for more complete analysis of Ab isotypes and their subclasses and kinetics (Fig. 1C). MCT and alum facilitated almost identical IgG, IgG1, and IgG2b responses. Only for IgG2a and IgG3 did 1 μg OVA-alum trigger a slightly earlier onset of the response than MCT. However, the end point responses after 6 wk were similar for MCT- and alum-based vaccines. At 100 μg OVA, all IgG kinetics and titers were comparable. OVA-specific IgE titers were lower with MCT than with alum at 100 μg (*p* = 0.015 by two-way ANOVA).

### MCT- and alum-based vaccines trigger comparable CD4 and CD8 T cell responses in mice

In order to facilitate the analysis of the effect of MCT and alum adjuvants on the stimulation of T cell responses, C57BL/6 mice were adoptively transferred lymphocytes from syngeneic OT-I and OT-II mice prior to immunization on days 0 and 7, with 100 μg OVA-MCT or OVA-alum. On day 14, spleens were harvested and analyzed by flow cytometry and ELISA for T cell proliferation and cytokine secretion.

**FIGURE 2.**
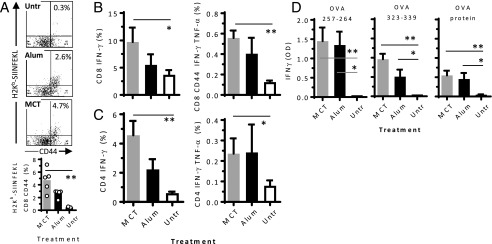
T cell response after MCT- or alum-adjuvanted immunization. C57BL/6 mice (*n* = 5) were adoptively transferred with OT-I and OT-II cells and immunized with 100 μg OVA on MCT (gray bars) or alum (black bars) 1 and 8 d later (individual mice are indicated). On day 15, spleen cells were analyzed by flow cytometry. (**A**) Dot blots and histograms show SIINFEKL-specific CD8 T cell activation (CD44) and proliferation (H2K^b^ pentamer). Frequencies of IFN-γ–producing and IFN-γ– and TNF-α–double-producing CD8 T cells (**B**) and CD4 T cells (**C**). (**D**) Splenocytes were also restimulated in vitro with MHC class I–restricted OVA aa 257–264 (SIINFEKL), MHC class II–restricted OVA aa 323–339, or OVA protein, and IFN-γ in culture supernatants was measured by ELISA. Mean ± SD of means are illustrated. The experiment is representative of two independent experiments with comparable results. *p < 0.05, **p < 0.01.

OVA-MCT and OVA-alum significantly stimulated proliferation of CD8 T cells specific for the H2K^b^-restricted OVA Ag OVA aa 257–264 (SIINFEKL) (Fig. 2A, *p* < 0.05 by Kruskal–Wallis). The percentages of IFN-γ–producing CD8 T cells and IFN-γ– and TNF-α–double-producing cells were higher in immunized mice than in untreated controls (*p* < 0.05), with no significant difference between MCT and alum (Fig. 2B). Similar effects were observed in CD4 T cells, with a positive effect of adjuvants (Fig. 2C). After restimulation of splenocytes in vitro with OVA protein or with MHC class I– or MHC class II–restricted OVA peptides, Ag-specific IFN-γ secretion from CD8 and CD4 T cells from immunized mice occurred (Fig. 2D), with no statistical difference between OVA-MCT and OVA-alum. C57BL/6 mice were also directly immunized to compare the capacity of the two adjuvants to trigger endogenous T cell responses (data not shown). Compared with nonimmunized mice, both OVA-MCT and OVA-alum induced significant and comparable CD8 T cell proliferation as well as IFN-γ and TNF-α production from CD8 and CD4 T cells.

### AIT with MCT-based vaccines reduced anaphylactic reaction in cat dander allergen-sensitized mice

**FIGURE 3.**
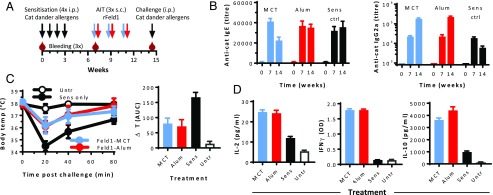
Immunotherapy of cat dander allergy. BALB/C mice (*n* = 5) were sensitized by weekly injections of cat fur allergen extract and received AIT with recombinant Fel d 1 allergen on alum (red) or MCT (blue) thrice s.c., as indicated (**A**). Finally, the mice were challenged with cat fur allergen extract to test tolerance to anaphylaxis. (**B**) Cat fur allergen-specific IgE (left) and Fel d 1–specific IgG2a (right) Ab titers were measured in sera before and after sensitization and AIT. (**C**) After challenge, changes in the body temperature were measured as an indicator for anaphylaxis. Left, Rectal body temperature as function of time after challenge. Right, Integrated AUC with baseline 38°C for the body temperature curves. (**D**) Splenocytes were restimulated with rFel d 1 for 20 h (IL-2) or 96 h (IFN-γ and IL-10), and cytokines were measured in the supernatants by ELISA. Abs are illustrated as mean ± SEM, and other results are illustrated as mean ± SD.

In order to test the relative strength of MCT and alum as adjuvants in allergy vaccines, the AIT efficacy was assessed in a mouse model of cat allergy (Fig. 3A). BALB/c mice were sensitized to cat dander allergens, then treated with recombinant Fel d 1 allergen mixed with MCT or alum. AIT with MCT, but not with alum, caused a decrease in cat-specific IgE antibodies (Fig. 3B). Fel d 1–specific IgG2a Ab titers increased after AIT and were comparable in MCT- and alum-treated mice. The titers were significantly higher than in sensitized control mice (*p* < 0.0.01). Four weeks after the third AIT shot, mice were i.p. challenged with cat dander allergen extract. Nonsensitized mice showed no reactions (Fig. 3C), whereas severe anaphylaxis was observed in sensitized mice having received no AIT. Anaphylaxis manifested as hypothermia within 20 min, with an average body temperature drop of 3.5°C (from 38.0 to 34.5°C). Moreover, hypothermia was accompanied by apathy, a hunched back, and piloerection. Sensitized mice that received AIT showed notably less signs of anaphylaxis, including significantly reduced hypothermia, as compared with mice that did not receive AIT (Fig. 3C). Within 60–80 min after challenge, treated mice recovered from the transient and moderate anaphylaxis, whereas sensitized animals still exhibited anaphylaxis symptoms and a temperature ∼2°C below the prechallenge level. When calculating the AUC for the body temperature drop, AIT with either MCT or alum adjuvants proved strongly protective (Fig. 3C, right panel; *p* = 0.01). No significant difference between alum- and MCT-treated mice was found. One week after the challenge, mice were euthanized and the splenocytes were restimulated with Fel d 1. AIT with alum and MCT equally resulted in significant IL-2, IFN-γ, and IL-10 secretion as compared with sensitized controls (Fig. 3D).

The efficacy of AIT with MCT was also performed in mice sensitized to birch pollen allergens (Table I). A cumulative allergen dose of 264 μg was split on five weekly or three biweekly injections. One group of mice received the five doses in a dose-increasing pattern. Upon AIT, allergen-specific IgG1 and IgG2a Ab responses were induced, especially after weekly AIT. Upon a systemic challenge with an allergen, sensitized controls reacted with severe anaphylactic hypothermia, whereas weekly AIT caused overall sufficient protection (Table I). The strongest protection was achieved after dose-increasing AIT.

**Table I.** Effect of MCT-based birch allergen AIT on specific Abs and tolerance to a birch allergen challenge

Readout	Number of AIT Sessions			
	No AIT	3	5	5-incr*^a^*
IgE	857	1183	1284	1256
IgG1 (× 10^5^)	10.9	10.3	21.4	18.9
IgG2a (× 10^3^)	1.7	9.3	15.4	16.9
Hypothermia, AUC*^b^*	1265	1292 NS	928 NS	437*

Mice (five to six per group) sensitized with birch pollen allergen extract received s.c. AIT with the same allergen adsorbed on MCT. The allergen dose of 264 μg birch pollen allergen (65.47 mcg Bet v 1) was split on three or five doses given with 14- or 7-d intervals. End point birch pollen allergen extract–specific Ab titers were analyzed by ELISA. Protection against anaphylaxis after a systemic challenge with birch pollen allergen was determined by measuring the change in body temperature upon challenge, and the temperature–time AUC was calculated with a baseline temperature of 38°C and with 4 h of assessment time.

^*a*^ The allergen dose given was increasing for each AIT session: 10 (2.48), 20 (4.96), 40 (9.92), 84 (20.83), and 110 (27.28) mcg with regard to total protein (Bet v 1 content in parentheses).

^*b*^ AUC was calculated for time–temperature curves after challenge with birch pollen allergen extract (baseline: 38°C, test time: 4 h).

* *p* < 0.01 as compared with no AIT.

NS, not significant as compared with no AIT (ANOVA).

### Allergic adverse events of AIT with MCT versus alum in a mouse model of anaphylaxis

**FIGURE 4.**
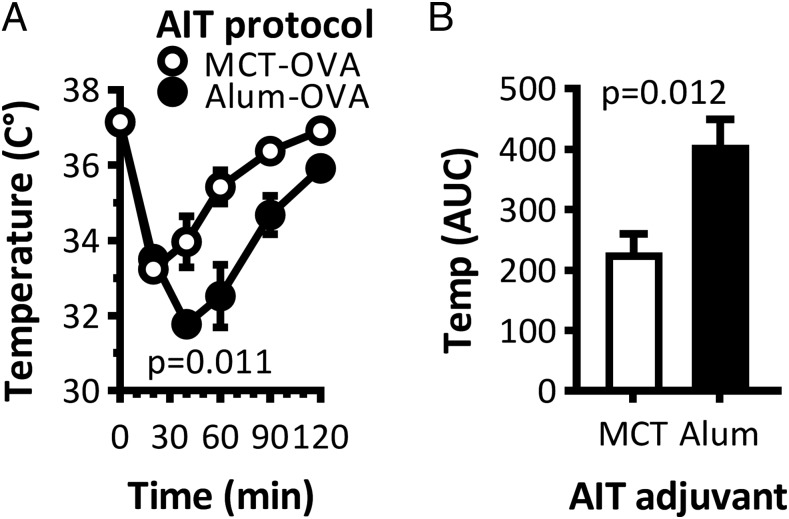
Safety testing of MCT- and alum-based vaccines in mice. BALB/C mice (*n* = 5) were sensitized by four weekly i.p. injections of OVA adsorbed on alum. Four weeks later, the mice received a single s.c. AIT with OVA on MCT (open circles and bars) or on alum (closed circles and bars). Body temperature changes are illustrated as a function of time after AIT (**A**) and integrated as AUC (**B**). The experiment is representative of two independent experiments with comparable results.

One of the side effects of AIT in allergic patients is the risk of local or systemic allergic reactions because of the allergen-containing AIT itself. To test the risk of anaphylaxis of AIT with MCT or alum, mice were sensitized to OVA as described above by four i.p. injections, then given a single s.c. AIT treatment with OVA. The body temperature in mice that received OVA-alum dropped from 37.5 to 31.8°C within 30 min (Fig. 4A), whereas OVA-MCT caused a drop to 33.5°C. When the AUC for the time–temperature function was calculated, AIT with MCT caused significantly (*p* = 0.012) less anaphylaxis than AIT with alum (Fig. 4B).

### The adjuvant properties of MCT are independent on TLR signaling

**FIGURE 5.**
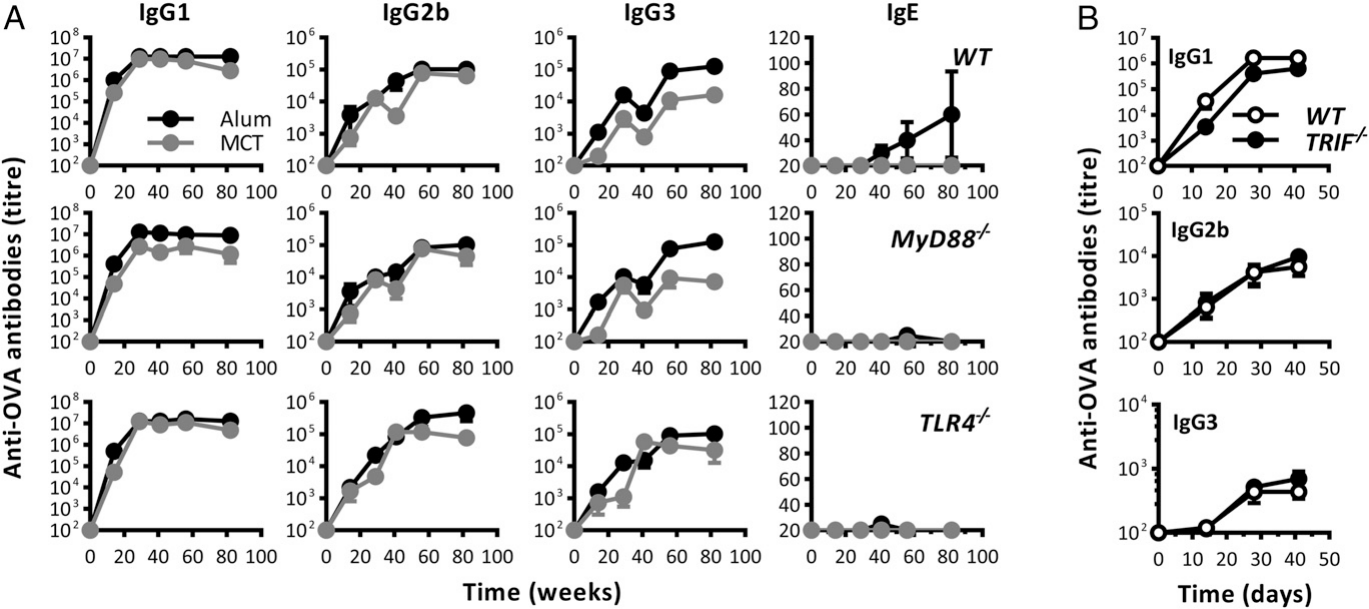
Immunogenicity testing in mice deficient in TLR signaling. (**A**) C57BL/6 wild-type mice as well as syngeneic MyD88- and TLR4-deficient mice were immunized with 40 μg OVA-MCT (gray circles) or 40 μg OVA-alum (black circles) on days 0 and 14. Abs in blood were measured by ELISA. (**B**) C57BL/6 mice as well as syngeneic TRIF-deficient mice were immunized with 10 μg OVA-MCT on days 0 and 14. Abs in blood were measured by ELISA. The experiment is representative of two independent experiments with comparable results and with four to six mice per group.

Whereas alum has been shown to act independently of TLR signaling, it is unknown if MCT used TLR for its adjuvant effects. To this end, TLR signaling–deficient mice were immunized twice with OVA on MCT or alum. The adjuvant effects of MCT or alum were not compromised in the absence of MyD88 signaling, as production of all IgG subclasses was stimulated by both OVA-MCT and OVA-alum (Fig. 5A). Alum, but not MCT, promoted anti-OVA IgE production in wild-type mice, whereas no IgE was observed in MyD88- or TLR4-deficient mice. Ag-specific IgG Ab responses were also not compromised in TRIF-deficient mice immunized with OVA-MCT (Fig. 5B).

### MCT induces caspase-dependent IL-1β secretion in vitro

**FIGURE 6.**
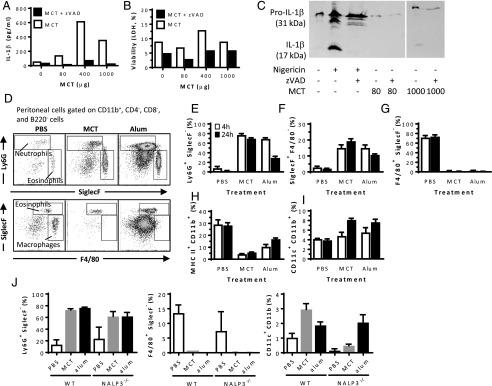
Assessment of inflammasome activation and other inflammatory reactions in vivo and in vitro. (**A**–**C**) THP-1 human monocytes were incubated with various doses of MCT in the presence or absence of zVAD. IL-1β secretion (A) and viability (B) were measured by cytokine and lactate dehydrogenase cytotoxicity ELISA, respectively, in supernatants. (C) Pro–IL-1β and cleaved IL-1β were separated by Western blot using nigericin as a positive control. For the sake of presentation, the blot was cropped and spliced as indicated with the vertical white line, excess sample replicates being omitted. (**D**) MCT, alum, or PBS were injected i.p. in mice, and cell populations in the peritoneal lavage were analyzed by flow cytometry as indicated. Infiltration of CD11b– and Ly6G–double-positive neutrophils (**E**), CD11b– and SiglecF–double-positive eosinophils (**F**), F4/80-positive macrophages (**G**), MHC class II– and CD11b–double-positive cells (**H**), and CD11c–CD11b double-positive DCs (**I**) were measured 4 h (open bars) and 24 h (closed bars) postinjection. The results with wild-type mice are representative of three independent experiments. (**J**) Percentage of neutrophils, macrophages, and DCs in wild-type versus NALP3 inflammasome knockout mice.

Because alum has been shown to activate the inflammasome, we investigated whether MCT also activated inflammasome. Firstly, human monocyte cells (THP-1) were incubated with various doses of MCT in the presence or absence of the pan-caspase inhibitor zVAD, which inhibits inflammasome-related cleavage of caspase-1 and subsequent maturation of pro–IL-1β to the secreted IL-1β. MCT caused secretion of IL-1β, and this was inhibited by zVAD, suggesting caspase dependency (Fig. 6A). IL-1β in the supernatant was not a result of MCT-induced cell death because cell death caused by MCT was not significantly different from untreated cells (Fig. 6B). As the IL-1β ELISA does not distinguish between pro–IL-1β and cleaved IL-1β, the culture supernatant was assessed by Western blot (Fig. 6C). In untreated THP-1 cells, only small amounts of pro–IL-1β were detected, whereas in MCT-treated cells, mature IL-1β was detected, and the secretion of the cleaved IL-1β was inhibited by zVAD. MCT- and alum-based vaccines caused similar acute but transient inflammatory responses.

One suggested adjuvant mechanism of alum has been the promotion of inflammatory responses at the injection site (9–11, 27); the adjuvant is injected i.p., and the inflammatory infiltrate is assessed by peritoneal lavage. Both alum and MCT caused infiltration of CD11b– and Ly6G–double-positive neutrophils (Fig. 6D, 6E) and of CD11b– and SiglecF–double-positive eosinophils (Fig. 6F), as assessed by flow cytometry. The effect of MCT lasted for at least 24 h. The effect of alum peaked at 4 h and decreased by 50% at 24 h. Moreover, resident F4/80-positive macrophages were reduced from 70% of all CD11b cells to <5% after administration of alum or MCT, and this effect lasted at least 24 h (Fig. 6G). Following the reduction of F4/80 macrophages in the peritoneal exudate cells, a marked drop in MHC class II– and CD11b–double-positive cells was also observed (Fig. 6H). A slight increase in CD11c–CD11b double-positive dendritic cells (DCs) was observed in the peritoneal exudate at 24 h postinjection (Fig. 6I). The inflammasome dependence of the inflammatory reactions in vivo was assessed in mice deficient for the NALP3 inflammasome (Fig. 6J). Alum or MCT were administered as above, and the inflammatory responses were analyzed 24 h postinjection. Neither peritoneal neutrophil nor F4/80 macrophage levels were affected by NALP3 deficiency. Although the count of CD11c DCs was lower in NALP3-deficient mice than in wild-type mice, the administration of MCT or alum increased the frequency of DCs in the peritoneal exudate.

### B and T cell responses to MCT- and alum-based vaccines are not dependent on or affected by inflammasome activation

**FIGURE 7.**
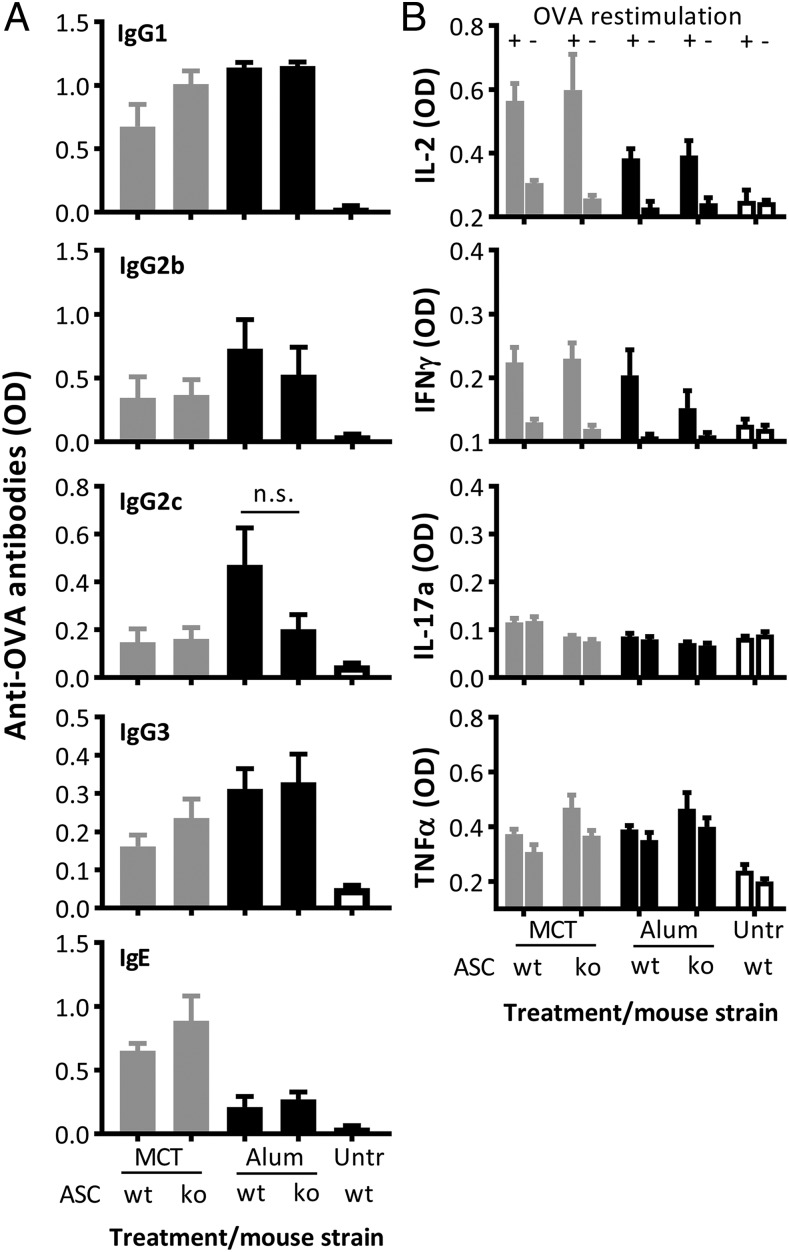
Analysis of the role of inflammasome activation on adjuvant mechanism of action of MCT and alum with regard to stimulation of B and T cell responses. Wild-type C57BL/6 and ASC knockout mice (*n* = 5) were immunized on days 0, 14, and 26 with 0.1 μg OVA on MCT or alum. (**A**) Mice were bled on day 42, and OVA-specific IgG1, IgG2b, IgG2c, IgG3, and IgE Abs were measured by ELISA. The Ab results (mean + SD) are illustrated as OD measured at a serum dilution of 1:3000. (**B**) Mice were euthanized on day 48, and splenocytes were restimulated with OVA (+) or were not restimulated (−) for analysis of cytokine secretion by ELISA (mean + SD). The experiment was not repeated.

Whether inflammasome activation affects the production of Abs by B cells or the secretion of cytokines by T cells was tested in ASC-deficient mice, which have nonfunctional inflammasomes. Mice were injected on days 0, 14, and 26 with 0.1 μg OVA on MCT or alum. Neither MCT nor alum required inflammasome activation for its adjuvant effect regarding the stimulation of Ag-specific IgG or IgE Ab responses, as illustrated in sera from day 42 (Fig. 7A). When day-48 splenocytes were restimulated in vitro with OVA and cytokines were measured in the culture supernatants, the results further revealed that T cell–mediated IFN-γ and IL-2 secretion were not compromised in the absence of a functional inflammasome (Fig. 7B). Neither MCT nor alum triggered Ag-specific secretion of IL-17a or TNF-α (Fig. 7B).

## Discussion

Along with its nearly century-long application as an adjuvant in conventional childhood and seasonal vaccines, alum is well established in licensed AIT (28, 29), and it represents the current state of the art in further AIT development. The recent decades have seen many attempts to find alternatives to alum in AIT and vaccination. Especially in AIT, MCT has shown clinical potential (28–31). Whereas the adjuvancy of alum was first explained by its depot effect—prolonged immune exposure resulting in sustained Ab titers (13, 32)—the adjuvancy of MCT is less understood. In this article, we report the first direct comparative study, to our knowledge, of alum and MCT in AIT. Immunological properties and mechanisms of action of the adjuvants were studied in mice.

Our study demonstrates that both MCT and alum enhanced IgG Ab responses to different allergens, such as OVA, birch pollen, and cat dander. Although a single injection with alum-adjuvanted allergen was slightly more immunogenic than MCT-adjuvanted allergen, MCT induced comparable high and sustained IgG Ab titers when the injection was repeated. Multiple MCT injections produced less IgE than did alum-based vaccines, confirming earlier reports (33), and this immunological profile formed part of the rationale for the development of MCT-based AIT. By consequence, the allergen-specific IgE-to-IgG2 ratio was higher with MCT-based than with alum-based AIT. In humans, the IgE-to-IgG4 ratio is a biomarker for effective AIT (34). The lower IgE response with MCT may be explained by less IL-4 secretion or stronger secretion of counteracting IFN-γ, hence a less Th2-skewed immune response (35, 36). Although MCT may not be characterized as a Th1-polarizing adjuvant, experiments in mice suggested that MCT triggered stronger Th1-associated immune responses than alum, especially at higher Ag doses. Particularly, MCT facilitated the recruitment of DCs and elicitation of CD8 T cell responses with associated IFN-γ and TNF-α production. These data are in line with studies demonstrating protective efficacy in influenza and malaria mouse models (17–19) and are favorable for long-term clinical tolerance following AIT (37, 38). Other important biomarkers for the monitoring of AIT are regulatory T cells and IL-10 (34). In the current study, alum and MCT resulted in similar IL-10 secretion from lymphocytes in vitro. The cellular analysis also suggested that alum and MCT trigger comparable CD4 T cell responses, whereas MCT triggers stronger CD8 T cell responses associated with Th1. Most importantly, the current study confirmed the benefit of MCT in AIT, which caused reduction in allergen-specific IgE and increased IgG1 and IgG2a and protection in the murine model of allergic anaphylaxis.

Alum can activate specific innate signaling pathways such as the NALP3 inflammasome (9–11). We could also demonstrate that MCT caused inflammasome activation in vitro and that MCT and alum produced similar inflammatory responses in vivo. Both adjuvants were characterized by immediate exudation of neutrophils and eosinophils. The current study is the first, to our knowledge, to characterize such inflammatory responses for MCT. Although many innate reactions are important for the onset of adaptive immunity, the role of inflammasome activation in immunization and AIT has not been precisely defined. We therefore tested if the immunogenicity of MCT- and alum-based vaccines was affected in mice deficient for ASC, the key common adaptor protein in inflammasome activation. Neither Ag-specific IgE nor IgG Ab responses were ASC dependent, and similar results have been reported for alum in mice deficient in IL-1R (27) or NLRP3 (12). Hence, although alum and MCT may activate the inflammasome, this does not affect the adaptive immune response needed for Ab production in AIT.

Most modern adjuvants act on APCs through direct ligation of pathogen recognition receptors such as TLRs, retinoic acid–inducible RLRs, nucleotide-binding NLRs, C-lectin CLRs, and cytosolic DNA sensors (39). Alternatively, the pathogen recognition receptor activation could be a secondary reaction following an unknown inflammatory trigger. We demonstrated that neither MCT nor alum exerted its adjuvant properties via TLRs because Ab responses were not affected by preventing signal transmission through MyD88, TRIF, or TLR4. Robust Ab responses to alum-based vaccines were previously demonstrated in MyD88- and TRIF-deficient mice (40).

Hence, MCT shares many of the immunological properties of alum, albeit harboring different physicochemical properties, such as particle size, morphology, adsorption characteristics, and local pharmacokinetics (41). Alum is usually an oxyhydroxide salt (boehmite) with a positive surface charge at pH 7.4 and a spherical dimension in the nanometer range. Self-associating MCT form needle-like crystals in the micrometer range. The different isoelectric properties may also influence the Ag release from the adjuvants. Alum and protein Ags are typically associated by a ligand exchange (e.g., via hydroxyl groups) whereas π-chi interaction is the predominant mode of adsorption for MCT (41). Whereas MCT is biodegradable and metabolized with a half life of 48 h (42), alum is not biodegradable and may remain at the injection site for years (28, 33, 43). These properties may affect Ag trafficking and shape the immune responses.

Alum’s potency as an adjuvant is undisputed. However, new combinations of adjuvants may help tackle areas of unmet need in emerging diseases or improve efficacy of AIT. Because alum does not stimulate TLRs, combinations with TLR ligands have been used to shift the immune response toward Th1 and thereby shorten the duration of AIT. AIT with MCT and MPLA exists as a shorter-course therapy option in named-patient products and is subject to current phase II and phase III clinical studies in the United States and in Europe (28). Combinations of alum and MPLA (44) or MCT and MPLA (45) improved Th1 responses in mice and enhanced grass pollen IgG1 and IgG4 responses in humans while reducing IgE responses and the number of AIT injections (46, 47). Th1 skewing and favorable clinical responses were also observed following injections of purified ragweed allergen conjugated to CpG oligonucleotide (48), although long-term benefits could not be confirmed (32).

MCT is established in AIT (43, 49). However, gaps exist in comparative in-depth and direct mechanistic studies of MCT in AIT. Our study demonstrates that MCT has adjuvant properties, making it a good alternative to alum-based AIT. Of course, murine studies have limited value with regard to the clinical potential of a novel adjuvant in human AIT because they use rather unnatural sensitization methods and because mice are short of biological factors that are considered important in human AIT (e.g., IgG4) (50, 51). However, murine studies can contribute to the understanding of the mechanism by which a novel adjuvant acts. Hence, the fact that MCT could activate the inflammasome in vitro and that MCT stimulated robust Ag-specific B and T cell responses in vivo when used as an adjuvant but these responses came independently of signal transmission through the inflammasome or TLR should be implied in further design of MCT-aided vaccination or AIT. Nonetheless, AIT with MCT stimulated less IgE and IL-4 than AIT with alum. Both MCT and alum induced high and sustained IgG titers and protection against anaphylaxis in sensitized mice. Moreover, MCT reduced allergic adverse events when injected into sensitized mice. This suggests that MCT may be a suitable and flexible partner for a wide range of AIT allergens or other vaccines.
